# Supplementary material for: Myostatin is associated with the presence and development of acute-on-chronic liver failure
Source: JHEP Rep. 2023 Apr 14;5(8):100761. doi: 10.1016/j.jhepr.2023.100761 (PMC10405090; doi:10.1016/j.jhepr.2023.100761)
Supplement: Multimedia component 1 [file mmc1.pdf]

Myostatin is associated with the presence and development of acute-on-chronic liver failure

Astrid Ruiz-Margáin, Alessandra Pohlmann, Silke Lanzerath, Melanie Langheinrich, Alejandro Campos-Murguía, Berenice M. Román-Calleja, Robert Schierwagen, Sabine Klein, Frank Erhard Uschner, Maximilian Joseph Brol, Aldo Torre-Delgadillo, Nayelli C. Flores-García, Michael Praktiknjo, Ricardo U. Macías Rodríguez, Jonel Trebicka

Table of contents

Fig. S1...2

Fig. S2...3

Table S1...4

Table S2...5

Table S3...6

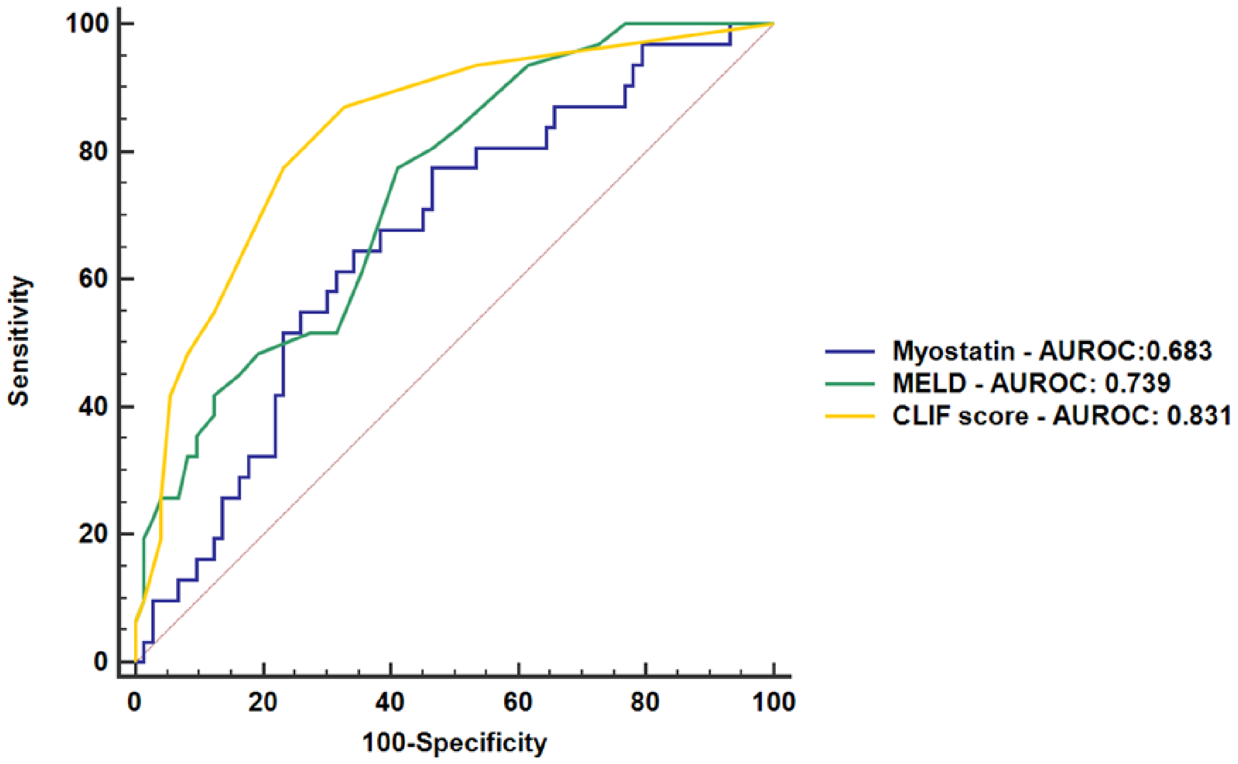

Fig. S1. Comparison of AUROC for myostatin, MELD and CLIF scores.

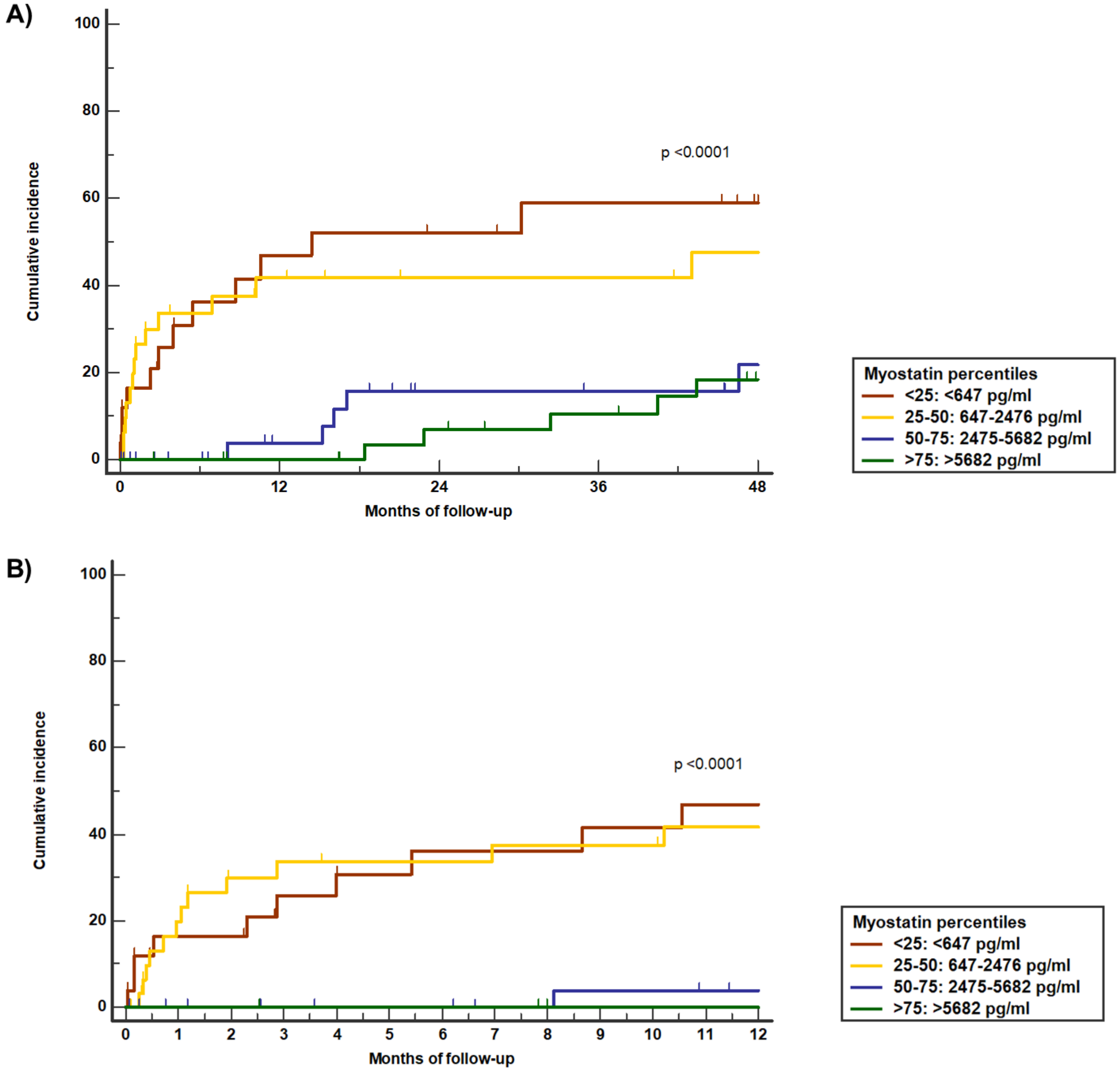

Fig. S2. Incidence of ACLF according to myostatin percentiles (A) and incidence of ACLF according to myostatin percentiles during first 12 months (B).

**Table S1. Main characteristics of patients according to ACLF grade**				
	**ACLF (n = 48)**	**ACLF 1 (n = 6)**	**ACLF 2 (n = 15)**	**ACLF 3 (n = 27)**
**Age (years)**	51 ± 15	51 ± 16	53 ± 11	49 ± 16
**Sex (F/M) (%)**	46/54	67/33	40/60	44.4/55.6
**MELD score**	20 ± 9	16.8 ± 3.7	18.3 ± 10	21.9 ± 9.3#
**MELD Na score**	21 ± 8	19.3 ± 4.2	19.8 ± 9.5	22.4 ± 8.3
**Myostatin (pg/ml)**	984 (278-2624)	1009 (194-4341)	1025 (281-3064)	960 (318-2483)
**PhA°**	3.8 (3.3-5.0)<br>5.0 ± 1.0	3.4 (2.7-4.1)<br>3.6 ± 1.0	4.4 (3.3-5)<br>4.3 ± 0.9	3.8 (3.3-5.1)<br>4.0 ± 1.1
**MAMC**	19.4 ± 3.2	17 ± 1.7*	19.1 ± 2.3	20 ± 3.6#
**Precipitants of acute decompensation (%)**				
**- Infection**	62.5	50	60	66.7
**- Bleeding**	14.6	33.3	6.7	14.8
**- No identified precipitant**	8.3	-	6.7	11.1
**- Other**	14.6	16.7	26.7	7.4
**LT n (%)**	10.4	1 (16.7)	4 (26.7)	0 (0)ǁ
**Death (%)**	79.2	5 (83.3)	10 (66.7)	23 (85.2)

Data presented as mean ± SD in variables with normal distribution, and median (p25-p75) in variables with non-normal distribution.
*Difference between groups ACLF1 vs ACLF2 ($p < 0.05$)
Difference between groups ACLF1 vs ACLF3 ($p < 0.05$)
ǁ Difference between groups ACLF2 vs ACLF3 ($p < 0.05$)
Student´ T test, Mann-Whitney´s U and Chi^2 test

Table S2. Characteristics according to sex		
	**Male (n = 69)**	**Female (n = 117)**
**Age (years)**	52.2 ± 11.2	54.2 ± 15.4
**Etiology of cirrhosis (%)**		
**-HCV**	24.6	35*
**- NASH / cryptogenic**	24.6	17.9
**- Autoimmune hepatitis**	5.8	16.2
**- Alcohol**	26.1	1
**- Primary biliary cholangitis**	2.9	13.7
**- Overlap syndrome**	5.8	9.4
**- Other**	10.2	6.8
**Child-Pugh (%)**		
**- A**	23.2	34.2 *
**- B**	30.4	35.0
**- C**	46.4	30.8
**Child-Pugh points**	8.5 ± 2.4	7.9 ± 2.5
**MELD score**	17 ± 9	13.5 ± 6 *
**MELD Na score**	19 ± 9	15 ± 7*
**Complications (%)**		
**- Ascites**	73.9	56.0*
**- Hepatic encephalopathy**	53.6	41.8
**Total bilirubin (mg/dL)**	2.7 (1.7-5.6)	2 (1.2-3.8)*
**Albumin (mg/dL)**	3.2 ± 0.8	3.2 ± 0.7
**Creatinine (mg/dL)**	1.4 ± 1.3	1 ± 0.7*
**INR**	1.4 ± 0.4	1.4 ± 0.4
**Sodium (mmol/l)**	136 ± 6	137 ± 6
**Myostatin (pg/ml)**	1574 (469-4915)	2913 (1021-5899)
**PhA°**	4.6 ± 1.2	4.6 ± 1.1
**MAMC**	21.7 ± 3.8	20.3 ± 4.2*
**HGS**	24 (13.8-28.5)	12 (8.6-16.6)*
**TSF**	18 (12-24)	21 (16.2-26)*
**ACLF (%)**	37.7	19.0*
**Acute decompensation (%)**	27.5	41.0
**Events of decompensation (%)**		
**1**	65	65
**2**	15	27
**3**	5	6
**4**	4	2
**LT (%)**	21.4	14.2
**Death (%)**	42	30.8

* $p < 0.05$

Data presented as mean ± SD in variables with normal distribution, and median (p25-p75) in variables with non-normal distribution. Student´ T test, Mann-Whitney´s U and Chi^2 test

**Table S3. Description of studies evaluating myostatin**			
	***Ruiz-Margáin A. et al.* (n = 186)**	***Nishikawa H et al.* (n = 198)**	***Skladany L et al.* (n = 355)**
Age	53.4 ± 14	67.5 (22.6–89.8)	56 (45-61)
Country	Mexico	Japan	Slovakia
Child-Pugh A/B/C (n)	56/62/68	123/72/3	-/155/199
Hand-grip strength<br>general<br>men/women	 14.3 (10-19.3)<br>24/12	 N/A<br>N/A	 N/A<br>28.5/17.6
Mid-arm circumference (cm)<br>general<br>men/women	 20.8 ± 4.1<br>21.7/20.3	 N/A<br>N/A	 N/A<br>27/25
Serum myostatin (pg/mL):<br>general<br>men/women<br>Child-Pugh A/B/C	2476 (647-5682)<br>1574/2913<br>5047/2269 /943	2994.4(578-12897)<br>3419.6/2662.4<br>2726/3615/3615	N/A<br>1959/1790<br>N/A
Mortality<br>general (%)<br>men/women (%)	 34.9<br>42/30.8	 N/A	 N/A<br>42.9/40.8
Follow-up (days)<br>general<br>men/women	 638<br>334/821	 1587<br>N/A	 N/A<br>248/256
Data presented as mean ± SD and median (p25-p75)			
