# Supplementary material for: Myostatin is associated with the presence and development of acute-on-chronic liver failure
Source: JHEP Rep. 2023 Apr 14;5(8):100761. doi: 10.1016/j.jhepr.2023.100761 (PMC10405090; doi:10.1016/j.jhepr.2023.100761)
Supplement: Multimedia component 4 [file mmc4.pdf]

Research article

JHEP Reports

Myostatin is associated with the presence and development of acute-on-chronic liver failure

Astrid Ruiz-Margáin,[1,2,3,†] **Alessandra Pohlmann**,[4,†] **Silke Lanzerath**,[4,†] Melanie Langheinrich,[5] Alejandro Campos-Murguía,[1] Berenice M. Román-Calleja,[1,2,3] Robert Schierwagen,[6] Sabine Klein,[6] Frank Erhard Uschner,[6] Maximilian Joseph Brol,[6] Aldo Torre-Delgadillo,[1] Nayelli C. Flores-García,[1] Michael Praktiknjo,[6,7] Ricardo U. Macías Rodríguez,[1,2,3,‡] Jonel Trebicka[6,7,*,‡]

[1]*Department of Gastroenterology, Instituto Nacional de Ciencias Médicas y Nutrición Salvador Zubirán, Mexico;* [2]*Liver Fibrosis and Nutrition Lab (LFN-Lab), Mexico;* [3]*MICTLÁN Network: Mechanisms of Liver Injury, Cell Death and Translational Nutrition in Liver Diseases-Research Network, Mexico;* [4]*Department of Internal Medicine I, University of Bonn, Bonn, Germany;* [5]*Department of Surgery, University of Greifswald, Greifswald, Germany;* [6]*Department of Internal Medicine B, University of Münster, Münster, Germany;* [7]*European Foundation for the Study of Chronic Liver Failure, Barcelona, Spain*

JHEP Reports **2023.** https://doi.org/10.1016/j.jhepr.2023.100761

Background & Aims: Acute-on-chronic liver failure (ACLF) has been linked to different pathophysiological mechanisms, including systemic inflammation and mitochondrial dysfunction. Sarcopenia has also been proposed as a potential mechanism; myostatin is a key factor inducing sarcopenia. Therefore, this study aimed to evaluate the association of myostatin levels with the development of ACLF and mortality in patients with cirrhosis.
Methods: We performed a prospective cohort study, including both outpatient and hospitalized patients with cirrhosis. Clinical, biochemical, and nutritional parameters were evaluated, and the development of acute decompensation (AD) or ACLF during follow-up was recorded. ACLF was defined according to the EASL-CLIF criteria. Receiver-operating characteristic, Kaplan-Meier and Cox regression analyses were performed.
Results: A total of 186 patients with the whole spectrum of cirrhosis were included; mean age was 53.4 ± 14 years, mean Child-Pugh score was 8 ± 2.5 and mean MELD score was 15 ± 8. There was a stepwise decrease in myostatin levels from a compensated stage to AD and ACLF. Myostatin correlated positively with nutritional markers and negatively with severity scores. The prevalence of sarcopenia was 73.6%. During follow-up, 27.9% of patients developed AD and 25.8% developed ACLF. Most episodes were grade 2-3, mainly (62.5%) precipitated by infections. The most common organ failures observed were in the liver (63.3%) and the kidney (64.6%). Receiver-operating characteristic analysis yielded <1,280 pg/ml as the best serum myostatin cut-off for the prediction of ACLF. In Kaplan-Meier curves and multivariate analysis, myostatin levels remained independently associated with the incidence of ACLF and survival.
Conclusions: There is a progressive decrease in myostatin levels as cirrhosis progresses, demonstrating an association of sarcopenia with the development of ACLF and increased mortality.
Impact and implications: Myostatin is a muscle hormone, it is decreased in patients with muscle loss and is a marker of impaired muscle function. In this study we show that myostatin levels are decreased in patients with cirrhosis, with lower levels in patients with acute decompensation and acute-on chronic liver failure (ACLF). Low myostatin levels in cirrhosis predict the development of ACLF and mortality independently of liver disease severity and sex.
© 2023 The Author(s). Published by Elsevier B.V. on behalf of European Association for the Study of the Liver (EASL). This is an open access article under the CC BY license (http://creativecommons.org/licenses/by/4.0/).

Introduction

Acute-on-chronic liver failure (ACLF) is distinct from acute decompensation (AD) and is characterized by the development of organ failure(s) (OFs) and high short-term mortality rates.[1] Apart from its more severe course, ACLF is also marked by a more pronounced systemic inflammatory response than seen in AD of cirrhosis.[2] Considering the high associated mortality and the lack of effective treatments, it is crucial to identify risk factors and predict the development of ACLF.

Malnutrition and specifically sarcopenia, the loss of muscle mass and decreased muscle function, are a common finding in chronic liver disease, and are related to the development of complications and mortality in patients with cirrhosis.[3,4] In this regard, the presence of sarcopenia measured by CT scan has been associated with the development of ACLF.[5] However, the underlying mechanism is not fully understood. One possible mechanism may be the increase in systemic inflammation and

Keywords: myostatin; malnutrition; sarcopenia; ACLF; cirrhosis; liver failure.
Received 2 August 2022; received in revised form 23 March 2023; accepted 5 April 2023; available online 14 April 2023
† Contributed equally to this work as first authors.
‡ Contributed equally to this work as senior authors.
* Corresponding author. Address: Department of Internal Medicine B (Gastroenterology, Hepatology, Endocrinology, Infectious Diseases), University Clinic Münster, University of Münster (WWU), Albert-Schweitzer-Campus 1, 48149, Münster, Germany. Tel.: +49 251 83 59689/57562; fax: +49 251 83 47570
E-mail address: jonel.trebicka@ukmuenster.de (J. Trebicka).

ELSEVIER

EASL The Home of Hepatology

mitochondrial dysfunction derived from malnutrition and sarcopenia, which, in turn, is involved in the development of ACLF.[2,6,7]

The muscle is an organ with immune regulatory properties. In fact, certain molecules produced in muscle, such as myokines, cytokines, and peptides including interleukin-6 (IL-6), possess immunomodulatory effects.[8] Myostatin, a key myokine involved in the regulation of skeletal muscle mass, has been shown to exert an immunomodulatory function in liver disease; myostatin deficiency was associated with decreased liver dysfunction and neutrophil infiltration into the liver, and improved bacterial clearance, leading to increased survival in a mouse model of sepsis.[9]

Specifically, higher baseline levels of IL-6 were associated with the development of ACLF.[2] However, IL-6 has pro-inflammatory and anti-inflammatory properties. While chronic exposure to pro-inflammatory cytokines, such as tumor necrosis factor-α, induce the pro-inflammatory properties of IL-6, physical exercise induces pulsatile IL-6 release as an anti-inflammatory response.[8]

Sarcopenia in cirrhosis is thought to be mediated, in part, by myostatin,[10] an autocrine inhibitor of skeletal muscle growth and development.[11] Myostatin levels are altered in patients with cirrhosis.[12] Interestingly, hyperammonemia upregulates the expression of myostatin via activation of the NF-κB pathway.[13] In contrast, follistatin, mainly secreted by the liver and involved especially in acute liver failure,[14] antagonizes myostatin, thus preventing it from executing its inhibitory effect on muscle growth.[15] In cirrhosis, this inhibitory effect of follistatin on myostatin is reduced.[16] The levels of myostatin may indicate sarcopenia in cirrhosis and since sarcopenia has been shown to be a risk factor for the development of ACLF, this study aimed to evaluate the role of myostatin levels and their association with the development of ACLF, and associated mortality, in patients with cirrhosis.

Patients and methods

This was a prospective cohort study carried out at a tertiary care center in Mexico City (Instituto Nacional de Ciencias Médicas y Nutrición Salvador Zubirán) from January 2014 to December 2019. It was conducted according to the principles in the Declaration of Helsinki and was approved by the local Institutional Review Board (REF.1347).

Patients were recruited in the outpatient clinic from the Liver Transplant Unit or the hospital ward; all patients provided their informed consent, and if they were unable to sign due to hepatic encephalopathy, their legal guardian signed the consent form on their behalf. Patients with a confirmed diagnosis of cirrhosis, who were older than 18 years of age were included in the study. The diagnosis of cirrhosis was based on the combination of clinical features, radiological imaging, presence of portal hypertension, compatible biochemical parameters, transient elastography (FibroScan), and/or liver biopsy. Patients with incomplete data were excluded from this study.

Follow-up

The time of established follow-up was from January 2014 to December 2019.

Outpatients and inpatients were invited to participate, all baseline evaluations were carried out, including blood sampling to obtain myostatin levels.

Patients were then followed with the aid of electronic clinical records, where events of AD or ACLF were recorded. None of the patients that were included we already in the disease severity category. All patients included were in different disease severity category then in the follow up.

The final groups in the study were created according to their decompensation status at the end of follow-up, *i.e.* no decompensation, AD or ACLF.

AD and ACLF

AD was defined as the presence of ascites (according to International Club of Ascites criteria) requiring hospitalization, hepatic encephalopathy (West Haven criteria) requiring hospitalization, primary bacterial peritonitis and/or variceal bleeding.

ACLF was defined based on the criteria described by the EASL-CLIF in the CANONIC study. The presence of organ failure(s) was defined after patients had received adequate management for 48 h following hospital admission. This timepoint (evaluation at 48 h) was established to avoid misdiagnosis of organ failures due to factors other than ACLF, *e.g.* hypovolemia-induced acute kidney injury (diuretics or variceal bleeding).

Myostatin determination

On the day of inclusion, a sample of peripheral blood was collected in tubes containing EDTA, which was immediately centrifuged. The obtained supernatant was stored at -80 °C and the levels of myostatin were determined using ELISA (# DY788-05, R&D Systems, USA) according to the manufacturer's assay guidelines. Briefly, serum and kit-provided standard samples were added to 96-well ELISA plates pre-coated with specific monoclonal antibodies and incubated for 3 h. Washing was performed four times, the specific enzyme-linked antibodies were added to each well. After an additional four washings, substrate solution was added to each well and incubated for 30 min. Subsequently, stop solution was added to stop the reaction. Plates were read at 450 nm by a microplate reader. A standard curve was prepared based on the serial dilutions data with concentration on the x axis (log scale) *vs.* absorbance on the Y axis (linear). The concentration of the sample was calculated from this standard curve.

Clinical and nutritional assessment

Clinical and biochemical parameters were collected from the electronic medical file to calculate Child-Pugh, model for end-stage liver disease (MELD), MELD-Na, Chronic Liver Failure – consortium (CLIF-C) ACLF and AD scores.

Nutritional markers were evaluated at the time of inclusion. Bioelectrical impedance-derived phase angle (PhA), mid-arm muscle circumference, triceps skinfold thickness (TST), and handgrip strength were measured.

Sarcopenia was evaluated using PhA, which is a nutritional marker obtained from bioelectrical impedance, widely validated in different diseases including cirrhosis and against the gold-standard CT-derived skeletal muscle index (SMI). It has 94% sensitivity for detecting sarcopenia when compared with SMI-diagnosed sarcopenia, both with comparable prognostic value.[17] PhA is easily obtained with a portable device and can be performed at bedside. This method was chosen due to ease of use and bioimpedance analysis to obtain PhA was performed using a mono-frequency device at 50 kHz (RJL systems Quantum IV). Bioimpedance analysis was performed after a 4-hour fasting

Table 1. Baseline characteristics of the study population.

	All (n = 186)
Age (years)	53.4 ± 14
Sex (F/M) (%)	(62.9/37.1)
Etiology of cirrhosis (%)	
HCV	31.2
NASH/cryptogenic	20.4
Autoimmune hepatitis	12.4
Alcohol	10.2
Primary biliary cholangitis	9.7
Overlap syndrome	8.1
Other	8
Child-Pugh (%)	
A	30.1
B	33.3
C	36.6
Child-Pugh points	8 ± 2.5
MELD score	15 ± 8
MELD-Na score	16 ± 8
Complications (%)	
Ascites	62.4
Hepatic encephalopathy	46.2
Myostatin (pg/ml)	2476 (647-5682)
Phase angle	4.7 (3.7-5.5)
Mid-arm muscle circumference	20.8 ± 4.1
Handgrip strength	14.3 (10-19.3)
Triceps skinfold thickness	20 (14-25.7)

MELD, model for end-stage liver disease; NASH, non-alcoholic steatohepatitis.
Data presented as mean ± SD for variables with normal distribution, and median (IQR) for variables with non-normal distribution.

period. With the patient in the supine position, four electrodes were placed, two on the right hand and two on the right foot, obtaining resistance (R), reactance (Xc) and PhA. Sarcopenia was diagnosed using PhA with previously validated cut-offs: <5.6° for males and <5.4° in females.[17]

TST was measured on the non-dominant arm to the nearest mm using a Harpenden caliper and mid-arm circumference was measured on the non-dominant arm to the nearest 0.1 cm with a non-stretchable measuring tape (Lufkin), the measurement was done at the mid-point between the olecranon and the acromion. Mid-arm muscle circumference (MAMC) was calculated from MAC and TST by the formula MAMC (mm) = MAC (mm) - (3.14 TST in mm). In patients with hepatic encephalopathy or sedation, both TST and MAMC were measured with the aid of a second person.

Handgrip strength (HGS) was measured with the patient seated using a Jamar handgrip dynamometer (Patterson Medical, Warrenville, Illinois). Measurements were made according to manufacturer's instructions and recorded to the nearest kg. To avoid bias, HGS was not performed when patients had overt hepatic encephalopathy. In some cases the test was able to be performed after hepatic encephalopathy resolution. Apart from clinical hepatic encephalopathy, covert hepatic encephalopathy was assessed in the patietns. When patients had a Number Connection Test-B (NCT-B test) result <220 s the test was considered reliable, as established by guidelines.[18]

Statistical analysis

Sample size estimation considered a hypothesized area under the receiver-operating characteristic curve (AUC) value of 0.65 (for myostatin levels), a non-discriminating AUC value (null hypothesis) of 0.5, and a proportion of negative/positive cases of 2/1. Finally, with an α error of 0.05 and a β error of 0.2, the final number was 43 positive cases and 86 negative cases, yielding 129 cases in total.

The Kolmogorov-Smirnov test was used to assess data distribution. Descriptive data is presented as absolute frequencies, mean ± SD or median (IQR). To compare two independent groups, Student's *t* test or Mann-Whitney *U* test were used. For comparison of three independent groups, ANOVA or Kruskal-Wallis tests were used as appropriate. To compare categorical variables, the Chi-squared test was used. Pearson's correlation analysis was performed to evaluate correlations between

Table 2. Comparison of baseline and final clinical and biochemical characteristics.

	Compensated (n = 86)		AD (n = 52)		ACLF (n = 48)	
	At inclusion	End of follow-up	At inclusion	End of follow-up	At inclusion	End of follow-up
Child-Pugh (%)						
A	50.6	24.4	13.5	5.8	10.4	0
B	30.6	46.5	40.4	23.1	31.3	2.1
C	18.8	29.1	46.2	71.2	58.3	97.9
Child-Pugh points	7 ± 2.4	7.9 ± 2.0**	9 ± 2	9.8 ± 1.6*	9 ± 2.4	11.5 ± 1.2**
MELD score	12 ± 6	15.3 ± 7**	16 ± 6	20.3 ± 7*	20 ± 9	31.1 ± 6**
MELD-Na score	13 ± 7	16.7 ± 7.1**	18 ± 7	21.4 ± 6.8*	21 ± 8	32.0 ± 6.9**
Total bilirubin (mg/dl)	2.2 (0.9-3.5)	2 (1.2-3.6)	2.4 (1.7-4.1)	3.2 (1.6-7.3)*	4.6 (2.1-19.7)	16 (6.8-28.8)**
Albumin (mg/dl)	3.5 ± 0.7	3.1 ± 0.7**	2.9 ± 0.7	2.6 ± 0.7*	3 ± 0.7	2.8 ± 0.8
Creatinine (mg/dl)	0.7 (0.6-0.9)	0.8 (0.6-1.0)	0.8 (0.7-1.4)	0.9 (0.7-1.3)	1.2 (0.7-1.8)	2.1 (1.4-3.2)**
INR	1.2 (1.1-1.3)	1.2 (1.1-1.5)	1.3 (1.2-1.6)	1.4 (1.2-1.6)	1.4 (1.3-1.7)	1.9 (1.5-2.4)**
Sodium (mmol/L)	137 ± 5	135 ± 5	135 ± 7	132 ± 6.5	137 ± 6	134 ± 7.4
Leukocytes (x10^3/μl)	4.1 (3.0-6.0)	4.5 (3.1-6.2)	4.8 (3.2-6.6)	4.6 (2.6-7.5)	5.1 (2.8-7.8)	8.5 (4.7-16.8)**
Neutrophils (%)	63.4 ± 12.1	66.6 ± 14.6*	72.3 ± 13.8	74.6 ± 14.8	72.2 ± 15.4	84.2 ± 10.4**
Precipitants of decompensation (%)						
Infection			50		62.5	
Bleeding			35.8		14.6	
Unknown			6.6		8.3	
Other (*i.e.*, alcohol, surgery, etc)			7.6		14.6	
Liver transplant (%)	16.5		25		10.4	
Death (%)	18.8 #		21.2 ‖		79.2 #,‖	

ACLF, acute-on-chronic liver failure; AD, acute decompensation; INR, international normalized ratio; MELD, model for end-stage liver disease.
Data presented as mean ± SD for variables with normal distribution, and median (IQR) for variables with non-normal distribution.
Paired *t* test and Wilcoxon test, **<0.001, *<0.05. Chi-squared test.
Difference between groups compensated *vs.* ACLF (p <0.05)
‖ Difference between groups AD *vs.* ACLF (p <0.05).

myostatin and study variables. To obtain cut-off values from myostatin, receiver-operating characteristic (ROC) curves and the Youden index were used. The incidence of ACLF and survival were evaluated using Kaplan-Meier curves and Cox regression analysis. Statistical analysis and figures were created using SPSS v21 and MedCalc v19.1. See supplementary CTAT table for further details.

Results

Baseline and end of follow-up characteristics

The baseline characteristics of the total study population, which were obtained during the initial visit when blood samples were collected, are shown in Table 1. The mean age was 53.4 ± 14 years, most patients were females (62.9%), the primary causes of liver disease were HCV infection, non-alcoholic fatty liver disease, and autoimmune liver diseases. The high number of autoimmune and cholestatic diseases is due to the focus of our hospital, which is a referral center for autoimmune and cholestatic diseases. The mean Child-Pugh score was 8 ± 2.5 points, and the mean MELD score was 15 ± 8. There was an even distribution among the different Child-Pugh categories, with 30.1% classified as Child-Pugh A, 33.3% as Child-Pugh B, and 36.6% as Child-Pugh C. The median levels of myostatin in the overall population were 2,476 (647-5,682) pg/ml, and markers of nutritional status were mainly below normal ranges.

At the end of follow-up, once the outcomes were registered, the population was divided into three categories: those who remained compensated (46.2%), those who experienced AD (28%), and those who developed ACLF (25.8%). Table 2 shows the characteristics of these groups at baseline and at the end of the follow-up period.

As expected, a higher proportion of patients with Child-Pugh B and C were observed in the AD and ACLF groups, with a worsening of severity scores in each group at the final evaluation due to the progression of the disease. Biochemical variables associated with the severity of liver disease, as well as leukocytes and neutrophils, were higher in patients with ACLF.

Infection and bleeding were identified as the main precipitants of both AD and ACLF. Though the rate of liver transplantation was numerically higher in patients with compensated cirrhosis or AD than in the ACLF group, this difference was not statistically significant. Finally, the mortality rate was substantially higher in patients with ACLF (79.2%) compared to those with compensated cirrhosis (18.8%) and AD (21.2%).

Regarding the characteristics of patients with ACLF in this study, there was a male predominance, higher prevalence of alcohol-related cirrhosis and higher severity scores. The majority of ACLF cases were categorized as grade 2 or 3, with infections being the primary trigger in 62.5% of cases. Liver and kidney failure were the most observed organ failures, with 63.3% and 64.6% of patients experiencing these, respectively. The most frequent precipitant of AD was infection, and the main clinical presentation was hepatic encephalopathy and spontaneous bacterial peritonitis (Table S1).

Myostatin and sarcopenia

Patients were classified according to the presence of sarcopenia by PhA; characteristics can be found in Table 3. There was a marked difference in myostatin levels according to the presence of sarcopenia, being lower when sarcopenia was present; a graphical representation of this finding is presented in Fig. 1B. Patients with sarcopenia more often had decompensated disease and a worse prognosis, with fewer of them reaching transplant, and with a higher incidence of ACLF and mortality.

ACLF, sarcopenia and sex

The differences in clinical and biochemical variables as well as outcomes according to sex can be found in Table S2. ACLF was more common in men than in women (37.7% *vs.* 19%), while AD showed the opposite trend, being more common in women than in men (41% *vs.* 27.5%). In general, mortality (42% *vs.* 30.8%) and liver transplantation (21.4% *vs.* 14.2%) were more common in men than in women. Differences were observed in etiology, biochemical variables and severity scales as well as regarding the presence of ascites. However, no significant differences were observed in the reported outcomes, and there was no statistical difference between males and females in myostatin levels (p = 0.105).

Table 3. Baseline characteristics of the study population classified according to nutritional status.

	Sarcopenia (n = 137)	No sarcopenia (n = 47)
Age (years)	54 ± 14	50 ± 12
Sex (F/M) (%)	62.8/37.2	63.8/36.2
Child-Pugh points	9 ± 2.4	7 ± 2.5*
MELD score	16 ± 7	11 ± 4*
MELD-Na score	18 ± 8	12 ± 5*
Complications (%)	69.1	42.6*
Ascites	55.5	19.1*
Hepatic encephalopathy		
Myostatin (pg/ml)	1987 (463-4,448)	5505 (2,330-6,918)*
PhA°	4.1 ± 0.9	6.2 ± 0.5*
MAMC	20.2 ± 4.1	22.7 ± 3.7*
HGS	12.8 (7.9-17.3)	18.4 (13.3-26.4)*
TST	20 (18–24)	19 (13–20)
LT (%)	16.1	21.3
Death (%)	40.9	17.0*
ACLF (%)	30.9	10.6*

ACLF, acute-on-chronic liver failure; HGS, handgrip strength; LT, liver transplantation; MAMC, mid-arm muscle circumference; MELD, model for end-stage liver disease; PhA, phase angle; TST, triceps skinfold thickness.
Data presented as mean ± SD in variables with normal distribution, and median (IQR) in variables with non-normal distribution.
Student's t test, Mann-Whitney U and Chi-squared test.
* Difference between groups (p <0.05).

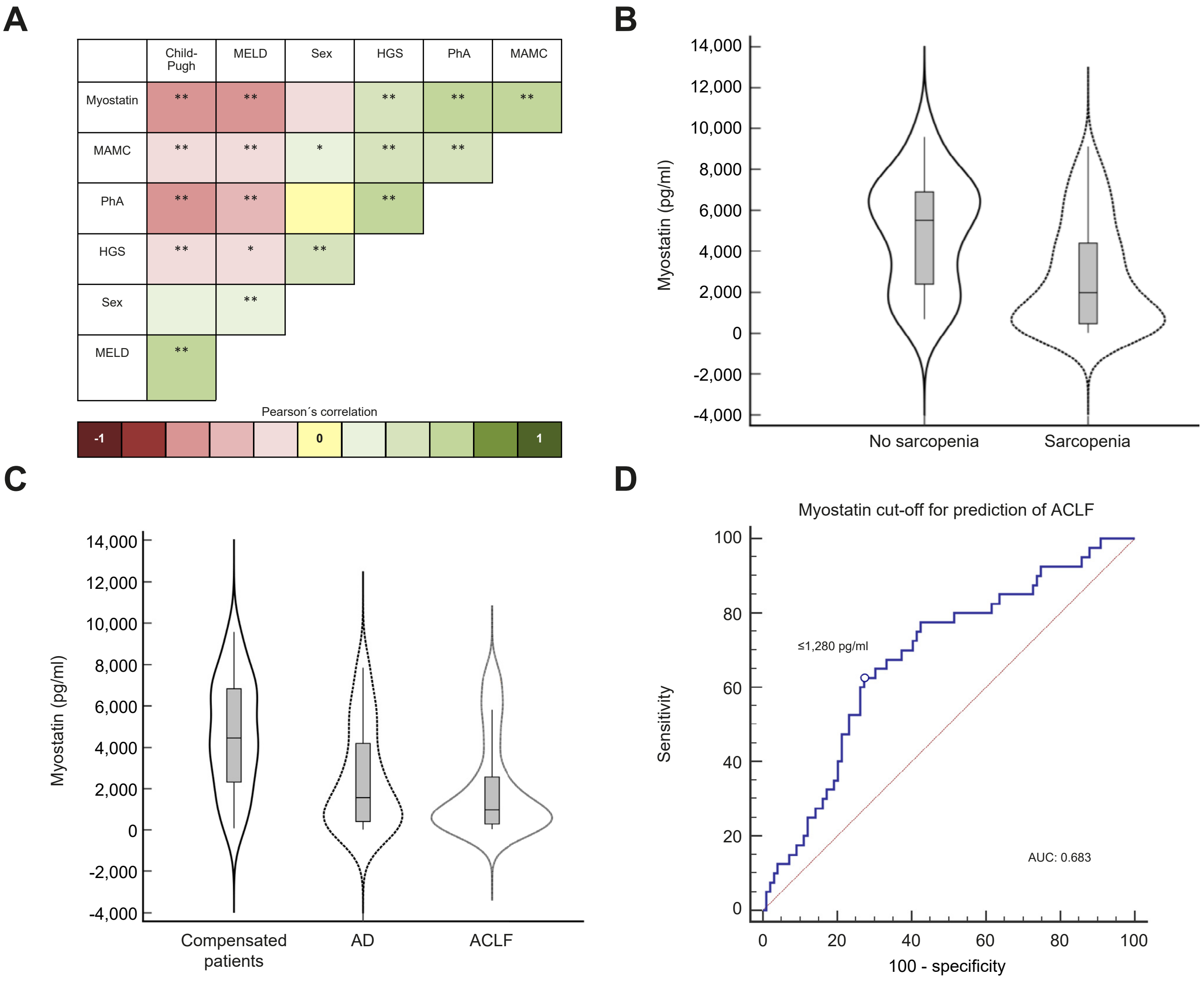

Fig. 1. Pearsońs correlation of myostatin with clinical and nutritional markers. (A) $^{**}p$ <0.001, $^{*}p$ <0.05 (Pearson's correlation coefficient r). (B) Levels of myostatin in patients with sarcopenia (1,987 [463–4,448]) and without sarcopenia (5,505 [2,330–6,918]) in the whole cohort. (C) Levels of myostatin in compensated patients (4,538.5 [2,274–6,850]), in patients with AD (1,587.5 [397–4,283]) and ACLF (984.5 [278–2,624]) in the whole cohort. (D) AUC of myostatin cut-off for ACLF prediction: 0.683, sensitivity: 62.5%, and specificity: 73.4%; standard error: 0.0497, 95% CI 0.597 to 0.758; *z* statistic: 3.651; significance level *p* (area = 0.5): 0.0003; Youden index J: 0.3523; associated criterion: ≤1,280 pg/ml. (ROC curve analysis and Youden index test). ACLF, acute-on-chronic liver failure; AD, acute decompensation; AUC, area under the receiver-operating characteristic curve; HGS, hand-grip strength; MAMC, mid-arm muscle circumference; MELD, model for end-stage liver disease; PhA, phase angle.

Myostatin and severity of liver disease

We explored correlations between myostatin and various nutritional and clinical variables (Fig. 1A). Myostatin levels positively correlated with nutritional markers and negatively with severity scores. There were no significant correlations between myostatin and age or sex.

Patients with compensated disease (Child-Pugh A) had higher levels of myostatin compared to those with decompensated disease (Child-Pugh B and C) (5,047 (3,864-6,869) pg/ml, 2,269 (538-6,190) pg/ml, and 943 (259-2,504) pg/ml, respectively, *p* <0.001). Furthermore, there was a gradient observed in the levels of myostatin when severity categories were compared, showing a decrease in myostatin levels as the severity of the disease increased (Fig. 1C).

Myostatin and population outcomes

ROC curves were constructed for myostatin levels to obtain the optimal cut-off value associated with the development of ACLF (Fig. 1D). The AUC for myostatin was 0.682 and the best cut-off was ≤1,280 pg/ml. The ROC curve for myostatin levels was compared with ROC curves for MELD and CLIF scores (Fig. S1), which exhibited AUCs of 0.739 and 0.831, respectively.

With the newly obtained cut-offs, Kaplan-Meier curves were created to evaluate the 6-month and total follow-up incidence of ACLF (Fig. 2A and B) and 6-month and total survival (Fig. 2C and D). The myostatin cut-off of ≤1,280 pg/ml was significantly associated with higher incidence of ACLF and more importantly, associated with decreased survival, with an approximate 2- to 3-fold decrease in cumulative survival between the groups. The

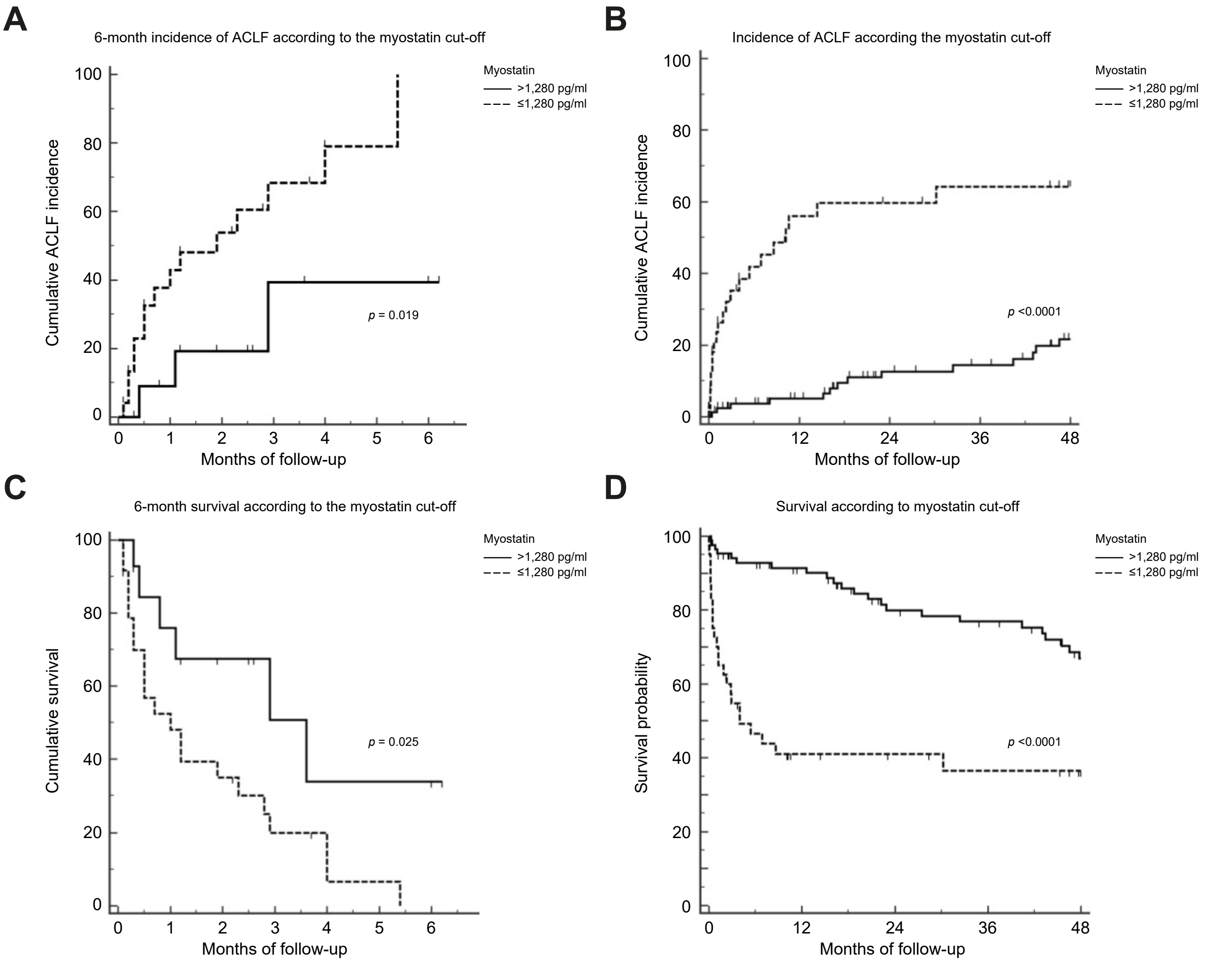

Fig. 2. 6-Month incidence of ACLF according to the cut-off of myostatin levels. (A) Months to incidence of ACLF: myostatin >1,280 pg/ml: 4.5 ± 0.8 *vs.* myostatin ≤1,280 pg/ml: 2.3 ± 0.4 (p <0.019) (n = 46) (Kaplan-Meier curves and log-rank test). (B) Incidence of ACLF in the total population according to cut-off of myostatin levels. Months to incidence of ACLF: myostatin >1,280 pg/ml: 61.97 ± 2.58 *vs.* myostatin ≤1,280 pg/ml: 22.21 ± 4.01 (p <0.001) (Kaplan-Meier curves and log-rank test). (C) 6-month survival according to the cut-off of myostatin levels. Mean survival in months: myostatin >1,280 pg/ml: 3.4 ± 0.7 *vs.* myostatin ≤1,280 pg/ml: 1.6 ± 0.2 (p <0.025) (Kaplan-Meier curves and log-rank test) (n = 46). (D) Survival in the total population according to the cut-off of myostatin levels. Mean survival in months: myostatin >1,280 pg/ml: 55.3 ± 2.93 *vs.* myostatin ≤1,280 pg/ml: 20.5 ± 3.75 (p <0.001) (Kaplan-Meier curves and log-rank test). ACLF, acute-on-chronic liver failure; AUC, area under the receiver-operating characteristic curve.

median follow-up in the total population was 21 (3-51) months. Also, to evaluate a gradation of effect of myostatin levels, we created four groups for myostatin based on the calculated percentiles of the cohort, <25th percentile, percentile 25-50, percentile 50-75 and >75th percentile, and we observed that those in the lower percentiles also had a higher incidence of ACLF compared to those in the higher percentiles (Fig. S2).

Finally, in the Cox regression analysis, the cut-off of myostatin ≤1,280 pg/ml remained independently associated with ACLF and survival when controlled for sex and MELD in the first model and sex and CLIF-C OF in the second model (Table 4).

Discussion

This study describes the association of myostatin with ACLF development; the manuscript aimed to provide evidence that muscle impairment, as indicated by myostatin levels, is associated with increased severity of cirrhosis, ACLF, and survival.

Myostatin was found to be positively correlated with muscle mass and function, as measured by PhA, MAMC, and HGS, and inversely correlated with severity scores. Additionally, in patients with sarcopenia, myostatin levels measured by PhA were significantly different from those in patients without sarcopenia.

These observations are important since the available evidence on serum myostatin and sarcopenia in chronic liver disease is conflicting.[19,20] Nishikawa *et al.* measured serum myostatin in stored sera of 198 hospitalized patients with cirrhosis, with a median follow-up period of 4.35 years. Lower levels of serum myostatin were associated with higher muscle mass measured by psoas muscle index.[19] In another study, Skladany *et al.* measured serum myostatin in 395 hospitalized patients with cirrhosis and controls, with a median follow up of 256 days and

Table 4. Variables associated with incidence of ACLF and mortality.

	HR (95% CI)	p value
**Univariable analysis for incidence of ACLF**		
Age	0.99 (0.97-1.01)	0.541
Sex	2.41 (1.36-4.29)	0.003
MELD	1.11 (1.08-1.15)	<0.001
Child-Pugh score	1.25 (1.14-1.36)	<0.001
Myostatin cut-off	6.31 (3.24-12.27)	<0.001
CLIF-C OF	1.61 (1.45-1.78)	<0.001
**Multivariable analysis for incidence of ACLF (Model 1)**		
Sex	2.16 (1.11-4.20)	0.022
MELD at end of follow-up	1.14 (1.09-1.19)	<0.001
Myostatin cut-off	6.19 (3.07-12.50)	<0.001
**Multivariable analysis for incidence of ACLF (Model 2)**		
Sex	1.41 (0.71-2.78)	0.321
CLIF-C OF	1.64 (1.44-1.87)	<0.001
Myostatin ≤1,280 pg/ml	3.64 (1.75-7.57)	<0.001
**Univariable analysis for incidence of mortality**		
Age	1.01 (0.99-1.02)	0.253
Sex	1.68 (1.03-2.75)	0.037
MELD	1.06 (1.03-1.10)	<0.001
Child-Pugh score	1.19 (1.10-1.29)	<0.001
Myostatin	3.92 (2.27-6.76)	<0.001
CLIF-C OF	1.02 (1.01- 1.03)	<0.001
**Multivariable analysis for mortality (Model 1)**		
Sex	1.46 (0.83-2.59)	0.190
MELD at end of follow-up	1.09 (1.05-1.12)	<0.001
Myostatin cut-off	3.28 (1.83-5.85)	<0.001
**Multivariable analysis for mortality (Model 2)**		
Sex	1.11 (0.61-2.01)	0.717
CLIF-C OF	1.29 (1.17-1.42)	<0.001
Myostatin ≤1,280 pg/ml	2.44 (1.35-4.49)	0.004

ACLF, acute-on-chronic liver failure; CLIF-C, Chronic Liver Failure – consortium; HR, hazard ratio; MELD, model for end-stage liver disease; OF, organ failure.
Cox regression analysis.

found that higher myostatin levels were correlated with higher muscle mass and better function, measured by MAMC, transversal psoas muscle index and HGS.[20] Arrieta *et al.* evaluated 112 older patients from long-term nursing homes, randomly assigned to a control or an intervention group, with a 6-month multicomponent exercise program, and measured serum myostatin levels. Higher concentrations of serum myostatin were found in fitter and less frail individuals than in frail ones. Also, a physical exercise intervention in men increased circulating myostatin concentrations.[21] Myostatin could act as a homeostatic regulator of muscle and therefore be associated with greater muscle mass and function.[21] These results are supported by the fact that muscle cells are the main site of myostatin production.[11] In our study, sarcopenia, assessed by multiple parameters, correlated with lower levels of myostatin, while the magnitude and range of circulating myostatin levels are similar to these data.

It is not only the correlation of myostatin with severity of sarcopenia that is important, but also its association with prognosis. Nishikawa *et al.* observed lower levels of myostatin in patients with Child-Pugh A compared to Child-Pugh B or C cirrhosis, as well as a sex specificity.[19] In contrast, median serum myostatin concentrations in men and women were similar in cirrhosis and only differed in patients without cirrhosis in our study, in agreement with the study by Skladany *et al.*, which was twice as large.[20] Importantly, in the latter study and similarly to our study, lower levels of myostatin were related to higher mortality.

In this study, we found that both ACLF and survival were related to lower levels of myostatin. Our initial objective was to establish a cut-off value for myostatin that could be further used in survival analysis. To achieve this, we conducted a ROC curve analysis and obtained a cut-off of ≤1280 pg/ml, with an AUC of 0.68, indicating good performance. However, as expected, when compared to more comprehensive severity scores such as MELD or CLIF, which incorporate multiple variables, myostatin had a relatively low performance. Nonetheless, it should be noted that our main aim was to establish a cut-off value for myostatin, rather than to identify a better performing marker.

In subsequent analysis, we created Kaplan Meier curves for the incidence of ACLF and overall survival, stratifying the analysis by this cut-off value. We evaluated this both in a short-term 6-month window with a smaller number of patients and a longer-term follow-up with a median follow-up time of 21 months in the total population. In both cases, we observed a significant difference in the incidence of ACLF and mortality which was higher in the group with lower myostatin values.

To evaluate the potential confounding effects of liver disease severity and sex, we constructed two multivariable models. And, after the inclusion of these variables, myostatin remained significantly associated with both ACLF and mortality.

Whether lower serum myostatin is the cause or a consequence of ACLF development and worsening of prognosis remains unclear; however, there are several hypotheses. One explanation is that, since muscle cells are the main site of myostatin production,[11] sarcopenia correlates with more advanced disease, which may lead to a decrease in myostatin production. The most convincing explanation is provided by the relationship of myostatin with inflammation. Both acute and chronic inflammation play an important role in myostatin regulation; for example, in an induced chronic arthritis rabbit model, animals developed markedly elevated serum C-reactive protein and muscle mass loss, while a significant reduction in both mRNA and protein expression of myostatin from gastrocnemii of rabbits was observed.[22] Further evidence on inflammation and downregulation of myostatin was reported by Åkerfeldt *et al.*, who measured myostatin in serum in two groups of patients scheduled for elective orthopedic and coronary bypass surgery. There was a significant decrease in myostatin concentrations in the post-surgical period, whereby the lowest myostatin concentration time point coincided with the highest systemic inflammation measured by C-reactive protein.[23] In this regard, patients with compensated cirrhosis, AD and ACLF have a different profile of systemic inflammation, being slightly abnormal, significantly increased, and highly abnormal, and showing "full-blown" inflammation during ACLF, respectively.[2] Therefore, if inflammation downregulates myostatin production, it is expected that as inflammation increases in the progression of compensated cirrhosis to AD and ACLF, myostatin levels would decrease. However, the exact mechanisms still need to be elucidated.

While the results of the present study suggest that lower levels of myostatin are associated with higher disease severity, conflicting results have been found in other studies, where both lower as well as higher levels of myostatin are found in patients with cirrhosis and advanced disease (Table S3).[24] This could imply that the muscle metabolism is highly dynamic, and it is dependent on the moment of assessment, where serial evaluations are warranted.

The strengths of this study are the inclusion of a cohort of patients before ACLF development with the full spectrum of cirrhosis (Child-Pugh A, B and C). This provides more information regarding myostatin levels during the crucial events in the natural history of cirrhosis. This study also has several limitations. First, a more detailed evaluation, showing how myostatin is regulated and varies over the course of ACLF with serial measurements in the same patient would have been most valuable. Furthermore, CT scans were not available, therefore we used PhA with very high sensitivity (94%) but low specificity for the assessment of sarcopenia; and finally, our center is a referral center with high prevalence of autoimmune and cholestatic liver diseases, with a greater prevalence in women; thus, our cohort might not reflect a representative distribution of cirrhosis etiologies for other centers. In addition, it is necessary to study the subjacent mechanism leading to changes in myostatin levels and its relationship with inflammation and other factors, including the role of different ILs (*e.g.* IL-6).

In conclusion, in our cohort of patients, lower levels of serum myostatin are not only associated with sarcopenia, but also with ACLF development and increased mortality. The implications of myostatin in decompensated cirrhosis should be further evaluated.

Abbreviations

ACLF, acute-on-chronic liver failure; AD, acute decompensation; AUC, area under the ROC curve; CLIF-C, Chronic Liver Failure – consortium; HGS, handgrip strength; IL, interleukin; MAMC, mid-arm muscle circumference; MELD, model for end-stage liver disease; OF, organ failure; PhA, phase angle; ROC, receiver-operating characteristic; TST, triceps skinfold thickness.

Financial support

This study was supported by the German Research Foundation (DFG) project ID 403224013 – SFB 1382 (A09), by the German Federal Ministry of Education and Research (BMBF) for the DEEP-HCC project and by the Hessian Ministry of Higher Education, Research and the Arts (HMWK) for the ENABLE and ACLF-I cluster projects. The MICROB-PREDICT (project ID 825694), DECISION (project ID 847949), GALAXY (project ID 668031), LIVERHOPE (project ID 731875), and IHMCSA (project ID 964590) projects have received funding from the European Union's Horizon 2020 research and innovation program. The manuscript reflects only the authors' views, and the European Commission is not responsible for any use that may be made of the information it contains. The funders had no influence on study design, data collection and analysis, decision to publish, or preparation of the manuscript.

Conflict of interest

The authors declare no conflict of interest. Jonel Trebicka has received speaking and/or consulting fees from Versantis, Gore, Boehringer-Ingelheim, Falk, Grifols, Genfit and CSL Behring. Please refer to the accompanying ICMJE disclosure forms for further details.

Authors' contributions

Study concept and design: ARM, RUMR and JT. Acquisition of research data: AP, SL, ML, ACM, BMRM, RS, SK, FEU, MJB, NCFG, MP. Analysis and interpretation of data: ARM, AP, SL, ML, ACM, BMRM, RS, SK, FEU, MJB, NCFG, MP, RUMR and JT. Drafting of the manuscript: ARM, AP, ACM, RS, RUMR, JT. Manuscript revision: all authors. Critical revision and editing: ARM, AP, ACM, RS, RUMR, JT. Funding acquisition: RUMR, JT.

Data availability statement

Data are available upon request.

Acknowledgements

The authors would like to thank the interns involved for their technical assistance.

Supplementary data

Supplementary data to this article can be found online at https://doi.org/10.1016/j.jhepr.2023.100761.

References

[1] Moreau R, Jalan R, Gines P, Pavesi M, Angeli P, Cordoba J, et al. Acute-on-Chronic liver failure is a distinct syndrome that develops in patients with acute decompensation of cirrhosis [cited 2017 May 15] Gastroenterol [Internet] 2013 Jun;144(7):1426–1437.e9. Available from: http://www.ncbi.nlm.nih.gov/pubmed/23474284.
[2] Trebicka J, Amoros A, Pitarch C, Titos E, Alcaraz-Quiles J, Schierwagen R, et al. Addressing profiles of systemic inflammation across the different clinical phenotypes of acutely decompensated cirrhosis. Front Immunol 2019 Mar;10.
[3] Hanai T, Shiraki M, Nishimura K, Ohnishi S, Imai K, Suetsugu A, et al. Sarcopenia impairs prognosis of patients with liver cirrhosis. Nutrition 2015;31(1):193–199.
[4] Ruiz-Margáin A, Macías-Rodríguez RURU, Ampuero J, Cubero FJFJ, Chi-Cervera L, Ríos-Torres SL, et al. Low phase angle is associated with the development of hepatic encephalopathy in patients with cirrhosis [cited 2017 May 16] World J Gastroenterol [Internet] 2016 Dec 7;22(45):10064. Available from: http://www.ncbi.nlm.nih.gov/pubmed/28018114.
[5] Praktiknjo M, Clees C, Pigliacelli A, Fischer S, Jansen C, Lehmann J, et al. Sarcopenia is associated with development of acute-on-chronic liver failure in decompensated liver cirrhosis receiving transjugular intrahepatic portosystemic shunt. Clin Transl Gastroenterol 2019 Mar;10(4).
[6] Ruiz-Margáin A, Pohlmann A, Ryan P, Schierwagen R, Chi-Cervera LA, Jansen C, et al. Fibroblast growth factor 21 is an early predictor of acute-on-chronic liver failure in critically ill patients with cirrhosis. Liver Transpl 2018;24(5).
[7] Daussin FN, Boulanger E, Lancel S. From mitochondria to sarcopenia: role of inflammaging and RAGE-ligand axis implication. Exp Gerontol 2021 Apr;146:111247.
[8] Nelke C, Dziewas R, Minnerup J, Meuth SG, Ruck T. Skeletal muscle as potential central link between sarcopenia and immune senescence, vol. 49. EBioMedicine. Elsevier B.V.; 2019. p. 381–388.
[9] Kobayashi M, Kasamatsu S, Shinozaki S, Yasuhara S, Kaneki M. Myostatin deficiency not only prevents muscle wasting but also improves survival in septic mice. Am J Physiol Endocrinol Metab 2021 Jan;320(1):E150–E159.
[10] Bunchorntavakul C, Reddy KR. Review article: malnutrition/sarcopenia and frailty in patients with cirrhosis. Aliment Pharmacol Ther 2020 Jan;51(1):64–77.
[11] McPherron AC, Lawler AM, Lee SJ. Regulation of skeletal muscle mass in mice by a new TGF-β superfamily member. Nature 1997 May;387(6628):83–90.
[12] García PS, Cabbabe A, Kambadur R, Nicholas G, Csete M. Elevated myostatin levels in patients with liver disease [cited 2018 Apr 27] Anesth Analg [Internet] 2010 Sep;111(3):707–709. Available from: http://www.ncbi.nlm.nih.gov/pubmed/20686014.
[13] Qiu J, Thapaliya S, Runkana A, Yang Y, Tsien C, Mohan ML, et al. Hyperammonemia in cirrhosis induces transcriptional regulation of myostatin by an NF-κB-mediated mechanism. Proc Natl Acad Sci U S A 2013 Nov;110(45):18162–18167.
[14] Lin T, Wang S, Munker S, Jung K, Macías-Rodríguez RU, Ruiz-Margáin A, et al. Follistatin-controlled activin-HNF4α-coagulation factor axis in liver progenitor cells determines outcome of acute liver failure [cited 2022 Feb 2] Hepatol [Internet] 2022 Feb 1;75(2):322–337. Available from: https://pubmed.ncbi.nlm.nih.gov/34435364/.
[15] Amthor H, Nicholas G, McKinnell I, Kemp CF, Sharma M, Kambadur R, et al. Follistatin complexes Myostatin and antagonises Myostatin-mediated inhibition of myogenesisMechanisms involved in the inhibition of myoblast proliferation and differentiation by myostatinMyostatin inhibits myoblast differentiation by down-regulating Myo. Dev Biol 2004;270(1):19–30.
[16] Rinnov AR, Plomgaard P, Pedersen BK, Gluud LL. Impaired follistatin secretion in cirrhosis. J Clin Endocrinol Metab 2016 Sep;101(9):3395–3400.

[17] Ruiz-Margáin A, Xie JJ, Román-Calleja BM, Pauly M, White MG, Chapa-Ibargüengoitia M, et al. Phase angle from bioelectrical impedance for the assessment of sarcopenia in cirrhosis with or without ascites [cited 2022 Feb 2] Clin Gastroenterol Hepatol [Internet] 2021 Sep 1;19(9):1941–1949.e2. Available from: https://pubmed.ncbi.nlm.nih.gov/32890753/.
[18] Lai JC, Feng S, Terrault NA, Lizaola B, Hayssen H, Covinsky K. Frailty predicts waitlist mortality in liver transplant candidates [cited 2017 May 11] Am J Transpl [Internet] 2014 Aug;14(8):1870–1879. Available from: http://www.ncbi.nlm.nih.gov/pubmed/24935609.
[19] Nishikawa H, Enomoto H, Ishii A, Iwata Y, Miyamoto Y, Ishii N, et al. Elevated serum myostatin level is associated with worse survival in patients with liver cirrhosis. J Cachexia Sarcopenia Muscle 2017 Dec;8(6):915–925.
[20] Skladany L, Koller T, Molcan P, Vnencakova J, Zilincan M, Jancekova D, et al. Prognostic usefulness of serum myostatin in advanced chronic liver disease: its relation to gender and correlation with inflammatory status. J Physiol Pharmacol 2019 Jun;70(3):357–368.
[21] Arrieta H, Hervás G, Rezola-Pardo C, Ruiz-Litago F, Iturburu M, Yanguas JJ, et al. Serum myostatin levels are higher in fitter, more active, and non-frail long-Term nursing home residents and increase after a physical exercise intervention. Gerontology 2019 Apr;65(3):229–239.
[22] Little RD, Prieto-Potin I, Pérez-Baos S, Villalvilla A, Gratal P, Cicuttini F, et al. Compensatory anabolic signaling in the sarcopenia of experimental chronic arthritis. Sci Rep 2017 Dec;7(1).
[23] Åkerfeldt T, Helmersson-Karlqvist J, Gunningberg L, Swenne CL, Larsson A. Postsurgical acute phase reaction is associated with decreased levels of circulating myostatin. Inflammation 2015 Aug;38(4):1727–1730.
[24] Bergen HR, Farr JN, Vanderboom PM, Atkinson EJ, White TA, Singh RJ, et al. Myostatin as a mediator of sarcopenia versus homeostatic regulator of muscle mass: insights using a new mass spectrometry-based assay. Skelet Muscle 2015 Jul;5(1).

Journal of Hepatology, Volume *5*

Supplemental information

Myostatin is associated with the presence and development of acute-on-chronic liver failure

Astrid Ruiz-Margáin, Alessandra Pohlmann, Silke Lanzerath, Melanie Langheinrich, Alejandro Campos-Murguía, Berenice M. Román-Calleja, Robert Schierwagen, Sabine Klein, Frank Erhard Uschner, Maximilian Joseph Brol, Aldo Torre-Delgadillo, Nayelli C. Flores-García, Michael Praktiknjo, Ricardo U. Macías Rodríguez, and Jonel Trebicka

Myostatin is associated with the presence and development of acute-on-chronic liver failure

Astrid Ruiz-Margáin, Alessandra Pohlmann, Silke Lanzerath, Melanie Langheinrich, Alejandro Campos-Murguía, Berenice M. Román-Calleja, Robert Schierwagen, Sabine Klein, Frank Erhard Uschner, Maximilian Joseph Brol, Aldo Torre-Delgadillo, Nayelli C. Flores-García, Michael Praktiknjo, Ricardo U. Macías Rodríguez, Jonel Trebicka

Table of contents

Fig. S1 2

Fig. S2 3

Table S1 4

Table S2 5

Table S3 6

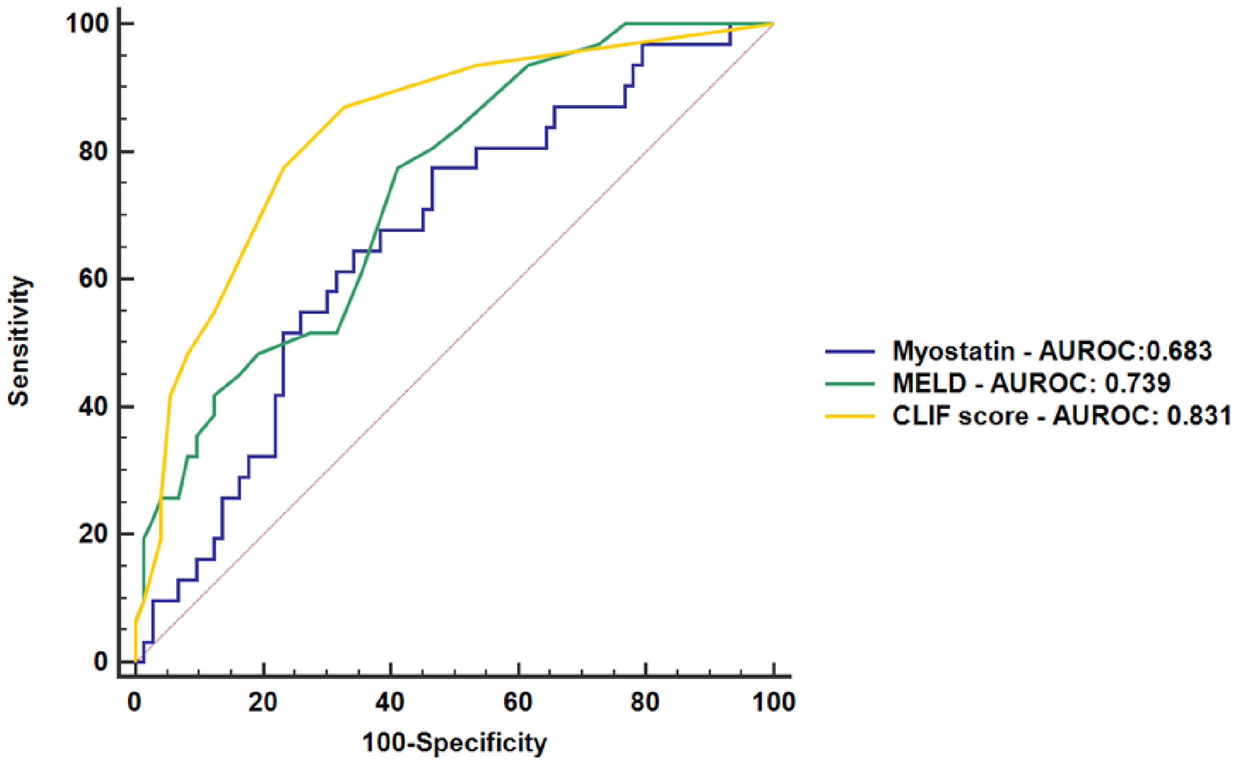

Fig. S1. Comparison of AUROC for myostatin, MELD and CLIF scores.

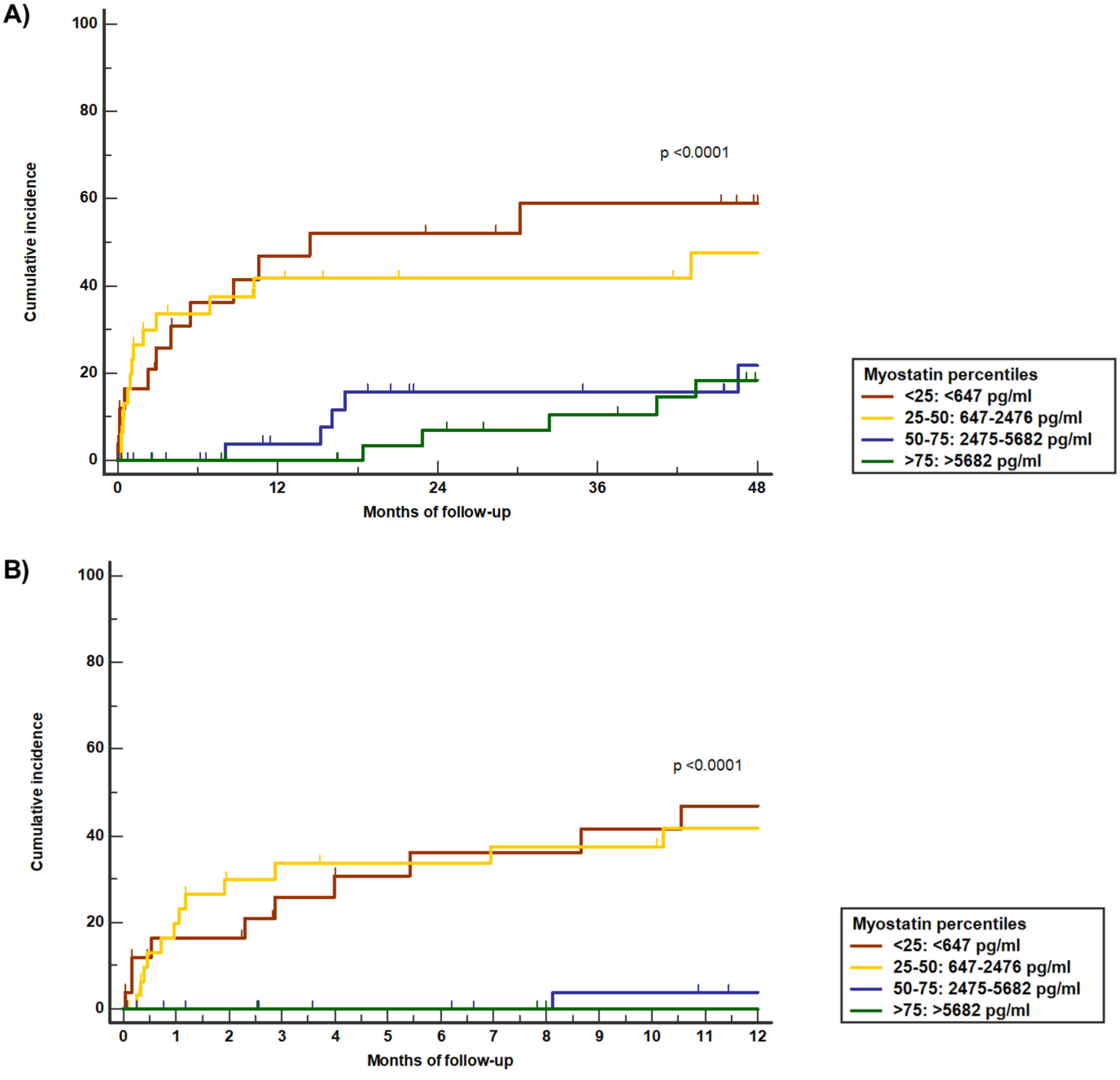

Fig. S2. Incidence of ACLF according to myostatin percentiles (A) and incidence of ACLF according to myostatin percentiles during first 12 months (B).

Table S1. Main characteristics of patients according to ACLF grade				
	**ACLF (n = 48)**	**ACLF 1 (n = 6)**	**ACLF 2 (n = 15)**	**ACLF 3 (n = 27)**
**Age (years)**	51 ± 15	51 ± 16	53 ± 11	49 ± 16
**Sex (F/M) (%)**	46/54	67/33	40/60	44.4/55.6
**MELD score**	20 ± 9	16.8 ± 3.7	18.3 ± 10	21.9 ± 9.3#
**MELD Na score**	21 ± 8	19.3 ± 4.2	19.8 ± 9.5	22.4 ± 8.3
**Myostatin (pg/ml)**	984 (278-2624)	1009 (194-4341)	1025 (281-3064)	960 (318-2483)
**PhA°**	3.8 (3.3-5.0)<br>5.0 ± 1.0	3.4 (2.7-4.1)<br>3.6 ± 1.0	4.4 (3.3-5)<br>4.3 ± 0.9	3.8 (3.3-5.1)<br>4.0 ± 1.1
**MAMC**	19.4 ± 3.2	17 ± 1.7*	19.1 ± 2.3	20 ± 3.6#
**Precipitants of acute decompensation (%)**<br>**- Infection**<br>**- Bleeding**<br>**- No identified precipitant**<br>**- Other**	 62.5<br>14.6<br>8.3<br>14.6	 50<br>33.3<br>-<br>16.7	 60<br>6.7<br>6.7<br>26.7	 66.7<br>14.8<br>11.1<br>7.4
**LT n (%)**	10.4	1 (16.7)	4 (26.7)	0 (0)ǁ
**Death (%)**	79.2	5 (83.3)	10 (66.7)	23 (85.2)

Data presented as mean ± SD in variables with normal distribution, and median (p25-p75) in variables with non-normal distribution.
*Difference between groups ACLF1 vs ACLF2 ($p < 0.05$)
Difference between groups ACLF1 vs ACLF3 ($p < 0.05$)
ǁ Difference between groups ACLF2 vs ACLF3 ($p < 0.05$)
Student´ T test, Mann-Whitney´s U and Chi^2 test

Table S2. Characteristics according to sex		
	**Male (n = 69)**	**Female (n = 117)**
**Age (years)**	52.2 ± 11.2	54.2 ± 15.4
**Etiology of cirrhosis (%)**		
**-HCV**	24.6	35*
**- NASH / cryptogenic**	24.6	17.9
**- Autoimmune hepatitis**	5.8	16.2
**- Alcohol**	26.1	1
**- Primary biliary cholangitis**	2.9	13.7
**- Overlap syndrome**	5.8	9.4
**- Other**	10.2	6.8
**Child-Pugh (%)**		
**- A**	23.2	34.2 *
**- B**	30.4	35.0
**- C**	46.4	30.8
**Child-Pugh points**	8.5 ± 2.4	7.9 ± 2.5
**MELD score**	17 ± 9	13.5 ± 6 *
**MELD Na score**	19 ± 9	15 ± 7*
**Complications (%)**		
**- Ascites**	73.9	56.0*
**- Hepatic encephalopathy**	53.6	41.8
**Total bilirubin (mg/dL)**	2.7 (1.7-5.6)	2 (1.2-3.8)*
**Albumin (mg/dL)**	3.2 ± 0.8	3.2 ± 0.7
**Creatinine (mg/dL)**	1.4 ± 1.3	1 ± 0.7*
**INR**	1.4 ± 0.4	1.4 ± 0.4
**Sodium (mmol/l)**	136 ± 6	137 ± 6
**Myostatin (pg/ml)**	1574 (469-4915)	2913 (1021-5899)
**PhA°**	4.6 ± 1.2	4.6 ± 1.1
**MAMC**	21.7 ± 3.8	20.3 ± 4.2*
**HGS**	24 (13.8-28.5)	12 (8.6-16.6)*
**TSF**	18 (12-24)	21 (16.2-26)*
**ACLF (%)**	37.7	19.0*
**Acute decompensation (%)**	27.5	41.0
**Events of decompensation (%)**		
**1**	65	65
**2**	15	27
**3**	5	6
**4**	4	2
**LT (%)**	21.4	14.2
**Death (%)**	42	30.8

* $p < 0.05$

Data presented as mean ± SD in variables with normal distribution, and median (p25-p75) in variables with non-normal distribution. Student´ T test, Mann-Whitney´s U and Chi^2 test

**Table S3. Description of studies evaluating myostatin**			
	***Ruiz-Margáin A. et al.* (n = 186)**	***Nishikawa H et al.* (n = 198)**	***Skladany L et al.* (n = 355)**
Age	53.4 ± 14	67.5 (22.6–89.8)	56 (45-61)
Country	Mexico	Japan	Slovakia
Child-Pugh A/B/C (n)	56/62/68	123/72/3	-/155/199
Hand-grip strength<br>general<br>men/women	 14.3 (10-19.3)<br>24/12	 N/A<br>N/A	 N/A<br>28.5/17.6
Mid-arm circumference (cm)<br>general<br>men/women	 20.8 ± 4.1<br>21.7/20.3	 N/A<br>N/A	 N/A<br>27/25
Serum myostatin (pg/mL):<br>general<br>men/women<br>Child-Pugh A/B/C	2476 (647-5682)<br>1574/2913<br>5047/2269 /943	2994.4(578-12897)<br>3419.6/2662.4<br>2726/3615/3615	N/A<br>1959/1790<br>N/A
Mortality<br>general (%)<br>men/women (%)	 34.9<br>42/30.8	 N/A	 N/A<br>42.9/40.8
Follow-up (days)<br>general<br>men/women	 638<br>334/821	 1587<br>N/A	 N/A<br>248/256
Data presented as mean ± SD and median (p25-p75)			
